# Ultrafast intersystem crossings in Fe-Co Prussian blue analogues

**DOI:** 10.1038/s41598-017-06664-4

**Published:** 2017-07-27

**Authors:** Michel van Veenendaal

**Affiliations:** 1 Department of Physics, Northern Illinois University, DeKalb, Illinois 60115 USA; 2 Advanced Photon Source, Argonne National Laboratory, 9700 South Cass Avenue, Argonne, Illinois 60439 USA

## Abstract

Ultrafast spincrossover is studied in Fe-Co Prussian blue analogues using a dissipative quantum-mechanical model of a cobalt ion coupled to a breathing mode. All electronic interactions are treated on an equal footing. It is theoretically demonstrated that the divalent cobalt ion reaches 90% of the $S{\boldsymbol{=}}\frac{{\bf{3}}}{{\bf{2}}}$ value within 20 fs after photoexciting a low-spin Co^3+^ ion by an iron-to-cobalt charge transfer. The doublet-to-quartet spin crossover is significantly faster than the oscillation period of the breathing mode. The system relaxes to the lowest manifold of divalent cobalt (^4^ *T*_1_) in 150–200 fs. Strong oscillations in spin-orbit coupling and the involvement of higher-lying quartets are found.

## Introduction

Ultrafast spin crossover is the intriguing phenomenon where the local moment of an ion is changed on the femtosecond timescale after irradiation with visible light. It has potential applications in magnetic data storage^1–4^. Spin crossovers result from the competition between the crystal field and higher-order terms in the Coulomb interaction. The crystal field causes a preferential occupation of certain orbitals. For example, a cubic crystal field splits the five-fold degenerate *d* orbitals into three- and two-fold degenerate states, denoted as *t*_2_ and *e*, respectively. For negatively-charged ligands, the *t*_2_ states are lower in energy. The Coulomb interaction, on the other hand, wants to increase the separation between the electrons, which can be achieved by maximizing the spin. The best-known examples are divalent iron complexes^1–5^, which show a transition from a low-spin ${t}_{2}^{6}$ (*S* = 0) configuration to a high-spin state (${t}_{2\uparrow }^{3}{t}_{2\downarrow }{e}_{\uparrow }^{2}$ with *S* = 2). The spin crossover requires intersystem crossings of *t*_2_ into *e* electrons. The absence of spin conservation indicates the crucial role of the spin-orbit interaction. Since electrons in *e* orbitals have their charge density pointing towards the ligands, the bond length increases significantly. Additionally, dissipative processes lower the energy from the photoexcited state to the high-spin state. Understanding the spin-crossover process is also important due to its use as a model for dissipative quantum-mechanical dynamics of many-body systems strongly interacting with the lattice. For example, spin crossovers were among the first spectroscopic studies performed at X-ray free electron lasers^6–9^. Due to the complexity of quantum-mechanical dissipation, the experimental developments are outpacing the theoretical progress. Here, the dynamics of the spin crossover following photoexcitation are calculated for a many-body system coupled to a damped breathing mode leading to a full relaxation to the (meta)stable state.

**Figure 1:**
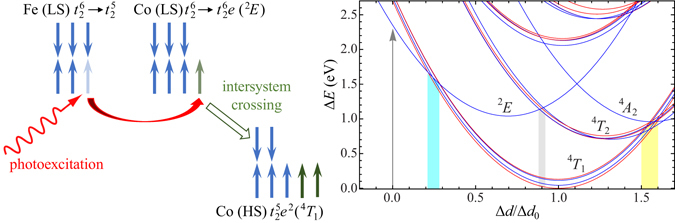
The left side shows a schematic depiction of the low-to-high spin crossover in Fe-Co Prussian blue analogues. After the photoexcitation of an electron from iron to cobalt, the divalent cobalt ion is in a low-spin ${t}_{2}^{6}e$ configuration denoted as ^2^ *E* in Mulliken notation. The system relaxes via an intersystem crossing to a high-spin ${t}_{2}^{5}{e}^{2}$ configuration (^4^ *T*_1_). The right side shows a semi-classical depiction of the energy levels of Co^2+^ with respect to the lowest state as a function of the normalized change in metal-ligand distance Δ*d*/Δ*d*_0_, where Δ*d*_0_ is the change between the lowest states of Co^2+^ and Co^3+^. In calculating this Figure, the breathing mode is assumed to be classical; the actual spin crossover is calculated using a quantum-mechanical breathing mode. The red and blue lines denote two- and four-fold degenerate states, respectively. The splitting of the parabolas is due to the spin-orbit interaction. The Mulliken notation is given for the states in absence of the spin-orbit splitting. The arrow indicates the initial photo excitation. The shaded regions denote relevant crossings between different levels.

Recent experiments on Fe-complexes have pushed the spin crossover switching time below 50 fs^5^. Since this is significantly smaller than the oscillation period of the breathing mode, it was speculated that high-frequency modes are essential in mediating the spin crossover^5^. Unfortunately, the iron spin crossover is complicated due to the need of two intersystem crossings. Also, the initial photoexcitation is a metal-to-ligand charge transfer and the involvement of the ligands adds another layer of complexity. In this Report, we therefore focus on the spin crossover in iron-cobalt Prussian blue analogues^10, 11^. The spin crossover is initiated by photoexciting an electron from Fe to Co. The absence of excitations involving the ligands^3–5, 12^ allows us to focus on the intersystem crossings on the transition-metal ion. In the ground state, both iron and cobalt have a low-spin ${t}_{2}^{6}$ configuration. The Fe ion becomes trivalent and remains low spin (${t}_{2}^{5}$), see the schematic diagram in Fig. 1. The configuration of the cobalt ion after photoexcitation is ${t}_{2}^{6}e$ with a total spin $S=\frac{1}{2}$, denoted as ^2^ *E* in Mulliken notation. A *t*_2_ → *e* intersystem transition causes a spin crossover leading to a high-spin ($S=\frac{3}{2}$) ${t}_{2}^{5}{e}^{2}$ configuration (^4^ *T*_1_)^10, 11, 13^. Furthermore, an increase in the cobalt-ligand bond length occurs in less than half an oscillation period for the spin crossover on cobalt^14^. The calculations provide insight into the processes of the ultrafast spin crossover. The full relaxation is split into two regions. An initial fast intersystem crossing between the doublet and quartet states occurs within 20 fs and is mainly determined by the time-development of the many-body Hamiltonian in the presence of a breathing mode. In the second region, a spillover into other quartet states temporarily decreases the occupation of the lowest quartet (^4^ *T*_1_). The damping of the oscillation of the breathing mode ensures the relaxation into ^4^ *T*_1_ in 100–200 fs. This is one of the first theoretical papers to show the time dependence of a complete relaxation of a low-to-high spin crossover using a realistic description of the local many-body electronic structure.

## Model

The Hamiltonian $\hat{H}={\hat{H}}_{{\rm{el}}}+{\hat{H}}_{{\rm{ep}}}$ consists of an electronic part ${\hat{H}}_{{\rm{el}}}$ and a term ${\hat{H}}_{{\rm{ep}}}$ describing the interaction between the 3*d* electrons and a breathing mode that changes the distance between the transition-metal ion and the surrounding ligands. The electronic part contains

1 $${\hat{H}}_{{\rm{el}}}=\sum _{{\mu }_{1}{\mu }_{2}{\mu }_{3}{\mu }_{4}}{U}_{{\mu }_{1}{\mu }_{2}{\mu }_{3}{\mu }_{4}}{d}_{{\mu }_{1}}^{\dagger }{d}_{{\mu }_{2}}^{\dagger }{d}_{{\mu }_{3}}{d}_{{\mu }_{4}}+\sum _{\mu \mu ^{\prime} }({{\rm{\Delta }}}_{\mu \mu ^{\prime} }+\zeta \langle \mu |{\bf{L}}\cdot {\bf{S}}|\mu ^{\prime} \rangle ){d}_{\mu }^{\dagger }{d}_{\mu ^{\prime} },$$

where ${d}_{\mu }^{\dagger }$ creates an electron in the 3*d* orbital with index *μ* = *mσ* with the *z* component of the angular momentum *m* = 2, 1, 0, −1, −2 and spin projection $\sigma =\pm \frac{1}{2}$. The first term on the right hand side contains the full multiplet *dd* Coulomb interaction. The parameters ${U}_{{\mu }_{1}{\mu }_{2}{\mu }_{3}{\mu }_{4}}$ can be expressed in terms of the radial integrals ${F}_{dd}^{2}=9.3$ and ${F}_{dd}^{4}\mathrm{=5.8}$ eV^15, 16^. The parameters for the Coulomb interaction are calculated within the Hartree-Fock limit and scaled down to 80% to account for intraatomic screening. The term Δ_*μμ*′_ causes the initial static cubic crystal field of 10*Dq* = 2.7 eV needed to describe the low-spin Co^3+^ ion. It splits the ten 3*d* levels into sixfold-degerate *t*_2_ states and fourfold-degenerate *e* states 10*Dq* higher in energy. The spin-orbit interaction *ζ* **L** · **S** has a strength *ζ* = 66 meV calculated within the Hartree-Fock limit. This many-body model has been very successful in describing spectroscopy on transition-metal compounds in the X-ray and optical region^15–19^. Note that, unlike other approaches^20–23^, there is no assumption of a small interaction (generally, the spin-orbit interaction).

The coupling between the electrons and the breathing mode is

2 $${\hat{H}}_{{\rm{ep}}}=\hslash {\omega }_{0}{a}^{\dagger }a-\sqrt{{\varepsilon }_{p}\hslash {\omega }_{0}}({a}^{\dagger }+a)\frac{\alpha +{\underline{n}}_{{t}_{2}}}{\alpha +1},$$

where *ħω*_0_ = 40 meV; *a* ^†^ is the step-up operator for the breathing mode and ${\underline{n}}_{{t}_{2}}$ is the number of *t*_2_ holes. The second term on the right-hand side is the electrostatic interaction between the electrons and the breathing mode. The metal-ligand distance increases for two reasons. First, there is a stronger repulsion of the ligands by electrons in th*e e* orbitals compared to *t*_2_ orbitals that have their lobes oriented towards and away from the ligands, respectively. This is accounted for by the ${\underline{n}}_{{t}_{2}}$ term and results in changes in metal-ligand distances for different Co^2+^ states. Secondly, there is an additional elongation due to the increase in charge on the transition metal after photoexcitation. This is included by the term *α* which accounts for the increase in metal-ligand distance between the low-spin state of Co^3+^ (Δ*d* = 0) and the lowest state of Co^2+^ (^4^ *T*_1_ with Δ*d*/Δ*d*_0_ = 1 and ${\underline{n}}_{{t}_{2}}=1$). The change in energy due to ${\hat{H}}_{{\rm{ep}}}$ given a number of *t*_2_ holes ${\underline{n}}_{{t}_{2}}$ is $-(\alpha +\langle {\underline{n}}_{{t}_{2}}\rangle \mathrm{)/(1}+\alpha ){\varepsilon }_{p}$. Since ${n}_{e}=1+{\underline{n}}_{{t}_{2}}$, the effective crystal field decreases as the numb*e*r of *e* electrons *n*_*e*_ increases, stabilizing the high-spin state. Both terms lead to an increase in the metal-ligand separation. The parameter values *ε*_*p*_ = 2.5 eV and *α* = 2 have been optimized to obtain a satisfactory agreement with *ab initio* calculations^11, 13^ with regards to energy separations and relative equilibrium positions. In the ground-state the cobalt ion is in a low-spin ${t}_{2}^{6}$ configuration. The system is photoexcited by a iron-to-cobalt charge transfer. For the model, this amounts to the addition of an electron to the cobalt ion creating a ${t}_{2}^{6}e$ configuration. The photoexcited state has a Franck-Condon spectrum with a maximum around *ε*_*p*_/*ħω*_0_ ≅ 63 excited vibronic levels. The calculation includes 300 excited vibronic levels, sufficient to linearize the vibronic dispersion for levels with significant occupation.

To obtain insight into the energy dependence of the states on the bond length, Fig. 1 shows the semi-classical limit. After the photoexcitation of an electron from iron to cobalt, the electron finds itself in the ^2^ *E* state. The photoexcitation occurs for Δ*d* = 0, see the arrow in Fig. 1, which is not the equilibrium position of the ^2^ *E* state. The high-spin state is ^4^ *T*_1_. The harmonic nature of the breathing mode leads to the parabolic dependence on the metal-ligand distance Δ*d*. Additional splittings occur in the parabolas due to the spin-orbit interaction. All the states are either twofold or fourfold degenerate. Note that the time-dependent calculations include the quantum-mechanical breathing mode with discrete energy levels.

The time dependence is calculated using a Schrödinger equation that includes the damping of the breathing mode described in more detail in ref. 24. The wavefunction at a particular time can be written as^24^

3 $$|\psi (t)\rangle =\exp \{-\,\frac{i}{\hslash }{\int }_{0}^{t}dt^{\prime} \,(\hat{H}+iD(t^{\prime} ))\}{d}_{e}^{\dagger }|{E}_{0}\rangle ,$$

where ${d}_{e}^{\dagger }$ creates an electron in an *e* orbital and |*E*_0_〉 is the low-spin ground state of Co^3+^. The time development contains two terms. First, there is the usual time development of the Schrödinger equation given by the Hamiltonian $\hat{H}$. This propagation is evaluated using Krylov subspace techniques^25^, where the exponent of the Hamiltonian for a particular time step is evaluated by constructing a tridiagonal matrix in a Lanczos basis using the wavevector from the previous time step as input. Diagonalizing the tridiagonal matrix leads to approximate eigenstates $|\tilde{m}\rangle$ that are used to evaluate the time dependence of the wavefunction. The second term *D*(*t*) includes the damping of the oscillation^24, 26, 27^. This term arises from the coupling of the breathing mode to a continuum of phonon states and integrating out these degrees of freedom. This effectively delocalizes the vibration of the breathing into the larger system, which damps the amplitude of the oscillation of the breathing mode. In the basis of eigenstates, this is given by *D*(*t*) = ∑_*m*_ *D*_*m*_(*t*)|*m*〉〈*m*| where

4 $${D}_{m}(t)=\frac{\hslash }{2}\frac{d\,\mathrm{ln}\,{P}_{m}(t)}{dt},$$

where *P*_*m*_(*t*) is the probability of finding the system in state *m* at time *t*. The time dependence of the probability is determined by the vibronic decay between the different eigenstates

$$\frac{d{P}_{m}}{dt}=-\sum _{m^{\prime}  < m}{|\langle m^{\prime} |a|m\rangle |}^{2}{\rm{\Gamma }}{P}_{m}+\sum _{m^{\prime}  > m}{|\langle m|a|m^{\prime} \rangle |}^{2}{\rm{\Gamma }}{P}_{m^{\prime} }.$$

Therefore, the decay process transfers probability to lower-lying states in an irreversible fashion. Therefore, although the wavefunction includes the effects of damping, the probability is a conserved quantity, *i.e*. 〈*ψ*(*t*)|*ψ*(*t*)〉 = 1. There are two important contributions to the decay. Inside a potential well, the matrix elements 〈*m*′|*a*|*m*〉 are dominated by the nonzero terms one would expect for a quantum harmonic oscillator with energies $E=n{\hslash }{\omega }_{0}+\frac{1}{2}$, *i.e*.$\,\langle n-1|a|n\rangle =\sqrt{n}$. These terms cause a damping of the amplitude of the oscillation inside a potential well. Due to the spin-orbit interaction and higher-order Coulomb terms, the values differ from $\sqrt{n}$ and there are additional couplings between the states, but the effects are still comparable to that of a damped quantum harmonic oscillator. Additionally, the matrix elements also cause a coupling between different potential wells due to the presence of the spin-orbit interaction and higher-order Coulomb terms, allowing further relaxation of the system.

For short time scales (*t* ≲ 500 fs), the system cannot be exactly diagonalized and the states are replaced by the approximate eigenstates $|\tilde{m}\rangle$. For long times scales (*t* ≳ 500 fs), only the lowest eigenstates have a finite occupation. Since the lowest eigenstates can be obtained using Arnoldi iteration, the exact results are used.

## Results

**Figure 2:**
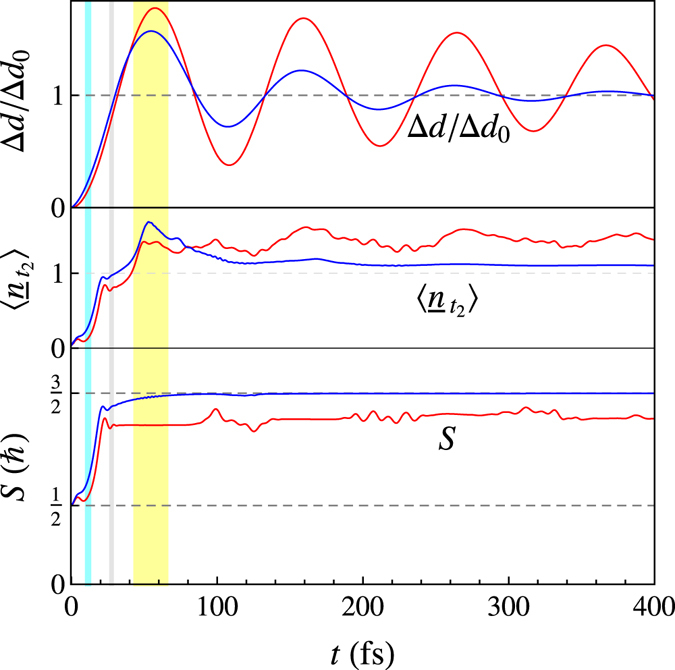
The time dependence of the expectation value of some important operators during the ultrafast spin crossover in a divalent cobalt ion. A comparison is made between the results without damping (red) and with a damping constant *ħ*Γ = 13 meV (blue). From top to bottom: the normalized displacement Δ*d*/Δ*d*_0_, the number of holes in the *t*_2_ orbitals $\langle {\underline{n}}_{{t}_{2}}\rangle =\langle {n}_{e}\rangle -1$, and the total spin *S*. The vertical lines indicate crossing of the energy curves in Fig. 1.

Figure 2 shows typical results obtained for the full quantum-mechanical Hamiltonian with and without damping of the breathing mode. We can distinguish two regions. First, an almost damping-independent ultrafast spin crossover occurs in the first 20 fs. After this, other quartet states become involved and the relaxation rate becomes damping-dependent. Let us first consider the former.

**Figure 3:**
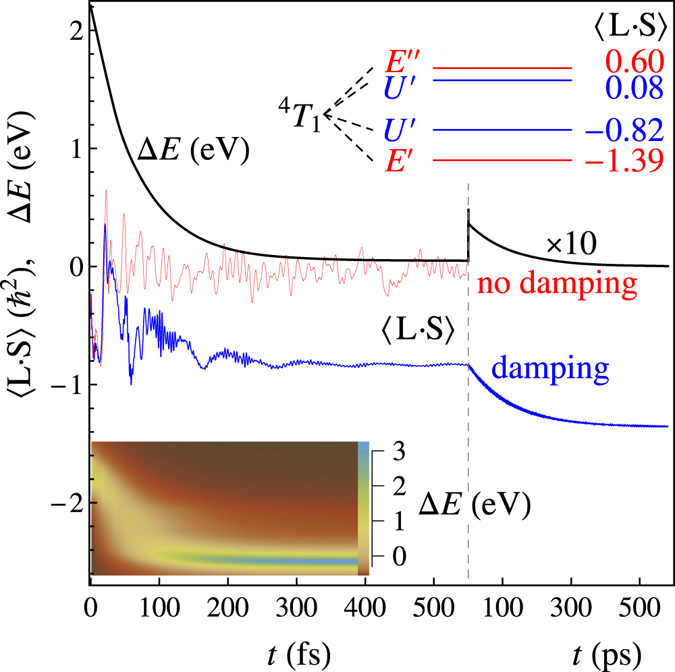
The time dependence of the energy difference Δ*E* with respect to the lowest state of divalent cobalt (black) and the expectation value of the spin-orbit coupling 〈**L** **·** **S**〉 (blue). The energy difference Δ*E* refers to the total energy of the local system, *i.e*. that of the electronic system and the breathing mode. Note the change in time scale for *t* > 550 fs. The calculations are done for a damping factor *ħ*Γ = 13 meV. For the spin-orbit coupling, a comparison is made with the calculation without damping (red). The schematic inset at the top shows how the lowest multiplet ^4^ *T*_1_ splits due to the spin-orbit interaction. The values of 〈**L** **·** **S**〉 are indicated. The inset at the bottom shows a density plot of the occupation as a function of Δ*E* (given on the right side of the inset) and *t* for the first few hundred femtoseconds. The color scheme for the density is given on the right side of the inset.

In the low-to-high spin crossover for Co^2+^, we expect *S* to increase from $S=\frac{1}{2}$ to $S=\frac{3}{2}$. In the calculation, the system reaches 90% of the value for a quartet in about 20 fs, see Fig. 2. This behavior is mirrored in the expectation value of the number of *t*_2_ holes. In a simple one-particle picture, one expects a transition from ${t}_{2}^{6}e$ to ${t}_{2}^{5}{e}^{2}$, *i.e*. $\langle {\underline{n}}_{{t}_{2}}\rangle$ should increase from 0 to 1, which is indeed reached in about 20 fs. This is very fast considering that the first maximum in the oscillation of the bond length only occurs at 55 fs. Additionally, the system has lost less than 25% of its energy, see Fig. 3. However, note that in the absence of damping, there is also a strong increase in *S*. The effects of damping in the doublet-to-quartet transition is relatively small. This demonstrates that the short time behavior is dominated by the development of the wavefunction by the Hamiltonian and that the damping of the breathing mode plays a minor role. For the first 10 fs, both *S* and $\langle {\underline{n}}_{{t}_{2}}\rangle$ change little. This corresponds to the motion of the wavepacket still consisting primarily of ^2^ *E* states. After the ^2^ *E* state crosses the ^4^ *T*_1_ states around 10 fs (see the vertical lightblue lines in Figs 1 and 2), we see a strong increase in the spin *S* and the number of *t*_2_ holes (and hence, the number of *e* electrons). From 10 to 20 fs, *S* increases from 0.68 to 1.35. This fast transition is a result of the dephasing of the doublet state into the Franck-Condon continuum of the quartet state. This gives approximately an exponential decay *e* ^−*γt*^ of the probability of the doublet state. The decay constant can be estimated by *γ* ∝ 2*πρζ* ^2^. The Franck-Condon factors follow a Poisson distribution. For ${\varepsilon }_{p}\gg \hslash {\omega }_{0}$, this approaches a Gaussian giving a density of states $\rho =\sqrt{\hslash \omega /(2\pi {\varepsilon }_{p})}$exp[−((*E* − *ε*_*p*_)/(2*εħω*_0_))^2^]. Approximating this by a simple square density of states at full-width-half-maximum *ρ* ≅ 1/*W* for $-\,\frac{W}{2}$ ≤ *E* ≤ $\frac{W}{2}$ with *W* = *ρ* ^−1^ = 2$\sqrt{2\,\mathrm{ln}\,2{\varepsilon }_{p}\hslash {\omega }_{0}}$ gives a dephasing time *γ* ^−1^ ≅ 18 fs, which is the right order of magnitude.

For *t* > 20 fs, the situation becomes more complex due to the involvement of other quartet states. The results also become dependent on the damping of the breathing mode. In the presence of damping, *S* steadily increases to the $S=\frac{3}{2}$. Note that in the absence of damping this value is not reached. The displacement Δ*d* reaches its first maximum around 55 fs and overshoots the equilibrium value for divalent cobalt by about 57%. The behavior of the expectation value of the number of *t*_2_ holes is peculiar. However, $\langle {\underline{n}}_{{t}_{2}}\rangle$ approaches a maximum of 1.7 around the same time as the maximum displacement is reached. This is a significant overshoot of the expected value for the ^4^ *T*_1_ state. We return to this below. The decay in energy is continuous, but significantly slower than the spin transition, see Fig. 3; 90% of the energy is dissipated via the phonon damping in 166 fs. The same trend can be observed from the occupations, see the inset in Fig. 3. After the photoexcitation, the system has a Franck-Condon distribution with a maximum around Δ*E* = 2.2 eV, where the energy difference is the combined energy of the electronic system and the breathing mode. The density then decreases steadily in energy up to 100 fs. Notice the large spread of eigenstates involved in the decay. Between 100–150 fs, the majority of the electron density is in the ^4^ *T*_1_ state, with some weak density around 1 eV. The expectation value of the spin-orbit coupling 〈**L** **·** **S**〉 shows dramatic fluctuations, Fig. 3, indicative of the involvement of the spin-orbit interaction in the spin crossover: dipping to −0.8 (in units of *ħ* ^2^) around 12 fs, only to increase to 0.4 at 21 fs, dropping again to −1 close to 60 fs. After some further oscillations, it appears to stabilize around −0.8.

In Fig. 3, although the system appears almost relaxed around 500 fs, it has in fact not reached the lowest state of Co^2+^. Although the wavefunction predominantly consists of ^4^ *T*_1_ states, there is still some density at higher energies. Additionally, the ^4^ *T*_1_ state is spin-orbit split, see the inset in Fig. 3. The splitting can be understood by taking the threefold orbital degeneracy as an effective angular momentum *L*_eff_ = 1 coupled to the $S=\frac{3}{2}$ spin giving ${J}_{{\rm{eff}}}=\frac{1}{2},\frac{3}{2},\frac{5}{2}$. Since the real angular momentum is opposite (**L** = −**L**_eff_), the lowest state has the angular and spin momentum coupled antiparallel. In octahedral symmetry, the ${J}_{{\rm{eff}}}=\frac{5}{2}$ splits further. This gives the irreducible representations *E*′ ⊕ *U*′ ⊕ *U*′ ⊕ *E*″, where *E* and *U* are two- and four-fold degenerate, respectively^15^. For these states, 〈**L** **·** **S**〉 = −1.39, −0.82, 0.08, and 0.60 and Δ*E* = 0, 43, 113, and 130 meV, respectively. The decay among these states takes a few hundred picoseconds, as evidenced by the further decrease of 〈**L** **·** **S**〉 and Δ*E* in Fig. 3. The lack of difference in equilibrium positions between the states explains the slow decay. Whereas the behavior in the first few hundred femtoseconds appears well explained by the model, additional scattering mechanisms (*e.g*., long-range Coulomb and spin scattering) cannot be ruled out for *t* > 1 ps.

**Figure 4:**
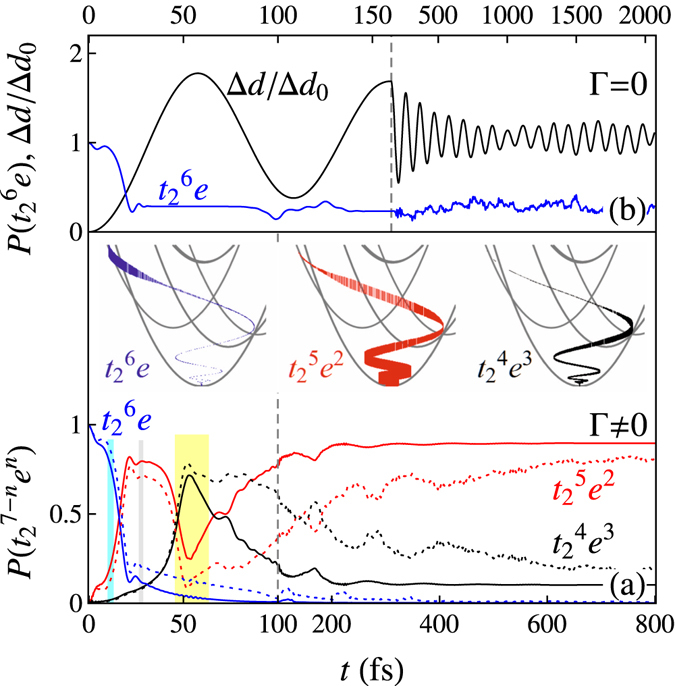
(**a**) The probability of finding the *t* ^6^ *e* (blue), ${t}_{2}^{5}{e}^{2}$ (red), and ${t}_{2}^{4}{e}^{3}$ (black) configurations as a function of time. The solid and dashed lines are for *ħ*Γ = 13 and 6 meV, respectively. The inset gives the displacement Δ*d* and energy Δ*E* in the energy level diagram from Fig. 1. The thickness of the line is proportional to the probability of finding the particular ${t}_{2}^{7-n}{e}^{n}$ configuration indicated in the diagram. (**b**) The normalized displacement Δ*d*/Δ*d*_0_ and the probability of finding the *t* ^6^ *e* configuration as a function of time in the absence of damping. Note the changes in time scales.

The same trends can be found by looking at the probabilities of finding the ${t}_{2}^{6}e$ and ${t}_{2}^{5}{e}^{2}$ configurations (corresponding predominantly to the ^2^ *E* and ^4^ *T*_1_ states, respectively), see Fig. 4(a). We see a strong increase in the latter configuration from 10–20 fs. At 29 fs, the ^2^ *E* and ^4^ *T*_2_ states cross, see Fig. 1 (indicated by the gray vertical lines). Around this time, the probability of finding the ${t}_{2}^{5}{e}^{2}$ configuration levels off and we observe an increase in finding the ${t}_{2}^{4}{e}^{3}$ configurations. The oscillation in the potential landscape is so strong that the system even reaches the point where the ^4^ *T*_2_ state drops below the ^4^ *T*_1_ state. There is even a small involvement of the ^4^ *A*_2_ states. This leads to a drop in the probability of finding ${t}_{2}^{5}{e}^{2}$ combined with a strong temporary increase in the probability of finding the ${t}_{2}^{4}{e}^{3}$ configuration, see the yellow vertical lines in Figs 3 and 4(a). When returning towards the equilibrium position (*t* > 60 fs) the damping of the oscillation ensures that the system settles in the ^4^ *T*_1_ state. Apart from a different timescale, the behavior for *ħ*Γ = 13 and 6 meV is qualitatively similar. Note that the probability of finding the ${t}_{2}^{4}{e}^{3}$ configurations does not approach zero. This is a many-body effect. The multiplet terms in the Coulomb interaction couple the ${}^{4}T_{1}[{t}_{2}^{5}{e}^{2}({}^{3}A_{2})]$ configurations to the ${}^{4}T_{1}[({t}_{2}^{4}{(}^{3}{T}_{1}){e}^{3}]$.

Although the calculation in absence of damping of the breathing mode describes the spin crossover in the first 20 fs, it fails to give a proper description of the further relaxation. Obviously, the energy remains constant, but additionally 〈**L** **·** **S**〉 oscillates around zero, see Fig. 3. At first, one might think that in the absence of damping the oscillation amplitude should remain constant, but this is not the case, see Fig. 4(b). The complexity of the energy landscape causes a dephasing of the oscillations leading to a decrease in the amplitude. Between 0.3–1 ps, the occupations oscillate around a constant value and no recurrence is observed (approximately 30, 60, and 10% probability of finding ${t}_{2}^{7-n}{e}^{n}$ with *n* = 1,2, and 3, respectively). Note that the probability of finding the ${t}_{2}^{6}e$ configuration does not approach zero, see Fig. 4(b).

## Conclusions

In this Report, the ultrafast spin crossover on the divalent cobalt site in Fe-Co Prussian blue analogues has been described using a dissipative Schrödinger equation^24, 26, 27^ for a Co^2^ ion coupled to a damped quantum-mechanical breathing mode. Although, similar physics could be described within a density-matrix framework^28–30^, these approaches are often restricted to simplified model systems. Although a detailed description of the electronic structure is possible within density-functional theory or quantum-chemistry, these methods do not always lend themselves well to dynamical calculations. Quantum-mechanical vibronic modes have only been included in the calculation of decay rates using Fermi’s golden rule^21–23^. Dynamics has been studied by treating the motion of the nuclei classically leading to Landau-Zener-type transitions between different states^20, 31–33^. However, these approaches have not yet been extended to systems where strong electron correlations dominate the physics.

The calculations show a spin crossover time of around 20 fs, significantly faster than the oscillation period of the breathing mode. This suggests a similar mechanism for the 50 fs spin-crossover time in Fe complexes that requires two *t*_2_ → *e* transitions^5^. Ultrafast time-dependent measurements of the low-to-high spin crossover on divalent cobalt ions are still sparse. Recent structural X-ray scattering experiments^14^ on [Co(terpy)_2_]^2+^ show that the Co-N bond length increases to its high-spin value in less than half the oscillation period of the breathing mode, in agreement with the calculation in Fig. 2. After a few oscillations, different pincerlike vibronic modes (that are not present in the Prussian-blue analogues) are excited and the oscillation amplitude of the breathing mode decreases. The delocalization of the oscillation is the primary source of the dissipation in the model^24^. This also demonstrates experimentally that the high-frequency modes are not crucial for the spin crossover, but are merely a result of the rapidly expanding bond lengths.

These calculations demonstrate that the Prussian-blue analogues are interesting compounds for time-dependent studies at X-ray free-electron lasers (XFEL). The calculations show strong variations in the expectation value 〈**L** **·** **S**〉. This quantity can be related to the branching ratio (the ratio ${I}_{{L}_{3}}/{I}_{{L}_{2}}$ of the integrated intensities of the *L*_2_ and *L*_2_ edges) of the isotropic *L*-edge x-ray absorption spectrum^34^. When 〈**L** **·** **S**〉 = 0, the branching ratio is expected to be the statistical value of 2 related to the degeneracies of the $j=\frac{3}{2},\frac{1}{2}$ values of the 2*p* core level. This value is reached around 30–50 fs. It also corresponds closely to the calculated value for a low-spin ^2^ *E* states. When the cobalt ion reaches the ^4^ *T*_1_ multiplet (aound 150–200 fs), the branching should have increased to around 3. Further relaxation among the spin-orbit split levels increases the branching ratio to around 5, which is the expected value for a high-spin Co^2+^ ion. These are substantial changes which should be clearly visible even with the reduced resolution often used in absorption experiments at XFEL’s. Therefore, although the effect on the metal-ligand bond lengths is smaller than in iron compounds, the stronger influence of the spin-orbit interaction on the spectral lineshape makes cobalt compounds more interesting from a spectroscopic point of view^17^.

The *K*-edge is not spin-orbit split and therefore not sensitive to changes in the spin-orbit coupling. However, it is more suitable to measure the splitting between the *t*_2_ and *e* levels and their respective populations. This allows one to probe the change in occupation following the spin crossover. Additionally, this technique should be sensitive in probing the overshoot into the ^4^ *T*_2_ and ^4^ *A*_2_ states around 50 fs.
